# Differential Influence of Early Life and Adult Stress on Urogenital Sensitivity and Function in Male Mice

**DOI:** 10.3389/fnsys.2017.00097

**Published:** 2018-01-09

**Authors:** Isabella M. Fuentes, Angela N. Pierce, Elizabeth R. Di Silvestro, Molly O. Maloney, Julie A. Christianson

**Affiliations:** Department of Anatomy and Cell Biology, University of Kansas Medical Center, Kansas City, KS, United States

**Keywords:** neonatal maternal separation, early life stress, pain, interstitial cystitis, chronic prostatitis/chronic pelvic pain syndrome, hypothalamic-pituitary-adrenal axis

## Abstract

Experiences of adverse childhood events have been associated with improper output of the hypothalamic-pituitary-adrenal (HPA) axis in adulthood, as well as development of comorbid functional pain disorders. Symptoms of chronic prostatitis/chronic pelvic pain syndrome frequently overlap with those of interstitial cystitis/painful bladder syndrome and symptom severity is often triggered by stress. The objective of this study was to investigate the influence early life stress and acute adult stress on (1) perigenital sensitivity, (2) micturition, (3) anhedonia, and (4) HPA axis regulation and output in male C56Bl/6 mice. Neonatal maternal separation (NMS) was performed for 3 h a day from postnatal day 1 to 21 and naïve pups remained unhandled during this time. As adults, male mice were tested for referred prostate sensitivity and micturition patterning prior to and 1 and 8 days after exposure to 1 h of water avoidance stress (WAS). Following testing, prostate and bladder tissues were used for mast cell and Western blot analysis and RT-PCR was performed on mRNA from hypothalamus, amygdala, and hippocampus. Serum corticosterone (CORT) was also measured by enzyme-linked immunosorbent assay (ELISA). A significant increase in perigenital sensitivity and micturition frequency was observed in NMS mice and these measures were exacerbated by WAS exposure. Exposure to NMS significantly increased mast cell degranulation in both the bladder and prostate. Mast cell degranulation was also increased in naïve prostate tissue following WAS exposure. Cytokine mRNA levels were influenced by both NMS and WAS exposure, though WAS had a larger impact on central gene expression. Protein levels of CRF_1_ were differentially regulated by NMS and WAS in the bladder and prostate and serum CORT levels were significantly diminished following stress exposure. Taken together, these data suggest that NMS results in neurogenic inflammation and hypersensitivity within the urogenital organs, coupled with diminished gene expression and output from the HPA axis. Future studies of NMS in male mice may provide a useful tool as a preclinical model of male chronic urological pain syndromes for investigating potential pharmacological and interventional therapies.

## Introduction

Stress has long been known to initiate or exacerbate symptoms associated with chronic pain disorders, including chronic prostatitis/chronic pelvic pain syndrome (CP/CPPS) and interstitial cystitis/painful bladder syndrome (IC/PBS). Childhood abuse or adversity can amplify the negative effects of stress, due to improper functioning of the hypothalamic-pituitary-adrenal (HPA) axis, and serve as a risk factor for developing mood and functional pain disorders later in life (Hu et al., 2007; Tyrka et al., 2008; Videlock et al., 2009; Riegel et al., 2014). In a community-based survey of men living in the Boston area, a reported history of abuse increased the odds ratio of having CP/CPPS symptoms and also raised both the pain and urinary scores using the National Institutes of Health chronic prostatitis symptom index (Hu et al., 2007). Another study of men and women migraineurs found a significant correlation between childhood maltreatment and increasing comorbid pain disorders, including IC/PBS (Tietjen et al., 2010).

Increased mast cell activation and/or degranulation is a common histological feature of biopsies from patients with CP/CPPS (Done et al., 2012; Murphy et al., 2014). Mast cells are highly granulated, stem cell-derived immune cells that contain numerous cytokines, proteases, histamine, and other potent algesic agents (Black, 2002). Mast cells can be activated to degranulate and release their stores of immune mediators by chemokines, such as monocyte chemoattractant protein 1 (MCP-1) and macrophage inflammatory protein 1α (MIP-1α) (Alam et al., 1992; Conti and DiGioacchino, 2001), as well as corticotropin releasing-factor (CRF) and urocortin 1 (Ucn1; family member of CRF) (Theoharides and Cochrane, 2004). As mast cells are often found in close proximity to nerves and their contents are known to act on nociceptive fibers, it is strongly asserted that mast cells play a key role in peripheral sensitization in chronic pain. As such, elevated histamine and mast cell tryptase levels have been detected in urine (Boucher et al., 1995; Erickson et al., 2002) and expressed prostatic secretion samples from CP/CPPS patients (Done et al., 2012). Furthermore, elevated nerve growth factor (NGF) levels in seminal plasma have been demonstrated to be directly correlated with pain severity (Miller et al., 2002; Watanabe et al., 2011). Elevated concentrations of NGF, histamine, and proinflammatory cytokines have been observed in IC/PBS patients’ serum (Jiang et al., 2013) and urine (Yun et al., 1992; Lotz et al., 1994; Jacobs et al., 2010; Corcoran et al., 2013). Mast cell infiltration and hypertrophy of sensory innervation has also been reported in biopsies from patients with IBS (Barbara et al., 2004, 2007; Akbar et al., 2008). Rodents exposed to NMS demonstrate increased growth factor and cytokine expression, including NGF, interleukin 6 (IL-6), IL-1β, IL-2, IL-4, IL-10, and interferon (IFN)-γ (Barreau et al., 2004a,b; O’Malley et al., 2011a,c), as well as infiltration of mast cells (van den Wijngaard et al., 2009, 2012; Lennon et al., 2013), in the distal colon, all of which can sensitize peripheral nociceptors and enhance visceral perception (Barreau et al., 2004a,b; Daniels et al., 2009; O’Malley et al., 2011a,c). Mouse models of experimental autoimmune prostatitis (EAP) develop pelvic mechanical allodynia, associated with increased mast cell infiltration and activation. However, mast cell deficient Kit^W-sh^/Kit^W-sh^ mice, as well as wildtype mice treated with mast cell stabilizers, do not develop pelvic mechanical allodynia following EAP (Done et al., 2012), further supporting the role of mast cells in disease etiology.

Mast cells also contain CRF, the primary initiator of the stress response in the HPA axis, as well as both receptor isoforms for CRF (CRF_1_ and CRF_2_), and can become activated upon exposure to stress (Black, 2002). Neonatal maternal separation (NMS) in rodents is a well validated model of early life stress and has been used extensively in rats as a model of irritable bowel syndrome (O’Mahony et al., 2011), and more recently in mice as a model of urogenital pain syndromes (Pierce et al., 2014, 2016; Fuentes et al., 2015). The objective of the current study was to investigate the impact of early life and adult stress on perigenital sensitivity, micturition, anhedonia, and central and peripheral aspects of HPA axis regulation and downstream output in male mice.

## Materials and Methods

### Animals

Experiments were performed on male C57Bl/6 mice (Charles River Laboratories, Wilmington, MA, United States) born and housed in the Research Support Facility at the University of Kansas Medical Center. Pregnant dams were delivered to the vivarium following the 2 weeks of gestation. It is possible transportation during pregnancy would pose a significant source of prenatal stress; however, there is evidence that between gestational days 16 and 22, maternal CRF receptor density and ACTH secretion to be reduced in response to daily intravenous administration of CRF in rats, presumably decreasing the likelihood of altering prenatal programming of the HPA axis in offspring (Neumann et al., 1998). Timing and duration of prenatal stress impose variable perturbations in offspring neurodevelopment (Welberg and Seckl, 2001). Typically, pregnant dams are in transit for 24–48 h, and are unhandled after delivery for a minimum of 72 h. Furthermore, all naïve and NMS litters were born of dams delivered in this manner, allowing us to be confident there would be no significant effect of acute prenatal stress on our outcomes. Mice were housed on a 12-h light cycle from 600 to 1800 h and received water and food *ad libitum*. All research performed conformed to the National Institute of Health Guide for the Care and Use of Laboratory Animals in accordance with the guidelines specified by the University of Kansas Medical Center Animal Care and Use Protocols. The animal use protocols (2013–2150, 2016–2344) were approved by the University of Kansas Medical Center Institutional Animal Care and Use Committee.

### Neonatal Maternal Separation

Date of birth was considered postnatal day 0 (P0). Beginning on P1, pups were removed daily from their home cages for 180 min (1100–1400 h), placed as litters in clean glass beakers with small amounts of home bedding material, and held in an incubator at 33°C and 50% humidity for 21 consecutive days (Fuentes et al., 2015). Naïve pups were unhandled outside of routine husbandry procedures. Naïve mice and mice that underwent NMS, termed NMS, were weaned on P22. Two separate cohorts were used to complete this study, one experimental and one control.

### Experimental Design

All experimental naïve and NMS males were subjected to acute water avoidance stress (WAS) as adults (>8 weeks of age) and assessed for perigenital sensitivity and micturition activity at 1-day pre-, 1-day post-, and 8-day post-WAS. Control naïve and NMS males were undisturbed in their home cages aside from normal husbandry procedures. *In vivo* experiments were conducted within the first 6 h of the light cycle. All *in vitro* analysis was performed on tissues from naïve and NMS mice that were not exposed to WAS (termed baseline) or sacrificed 8 days after WAS exposure.

### Water Avoidance Stress

Water avoidance stress was performed for 1 h, within the first 6 h of the light cycle, for 1-day. Mice were placed individually on a round platform (5 cm diameter) centrally affixed to the bottom of a container (36 cm length × 31 cm width × 27 cm height) filled with room temperature tap water up to 1 cm below the top of the platform.

### Behavioral Analysis

#### Von Frey Monofilament Testing

Perigenital sensitivity was assessed as previously described (Fuentes et al., 2015). Mice were first acclimated to the sound proof testing room in their home cages for 30 min prior to testing. Naïve and NMS mice were then placed inside individual clear plastic chambers (11 cm × 5 cm × 3.5 cm) on a wire mesh screen elevated 55 cm above a table. The up–down method was performed to test mechanical sensitivity using a standard set of von Frey monofilaments (1.65, 2.36, 2.83, 3.22, 3.61, 4.08, 4.31, 4.74 g; Stoelting Co., Wood Dale, IL, United States) (Dixon, 1980). Beginning with the 3.22 g monofilament, mice received a single application to the scrotum. A negative response was followed by the next larger filament and a positive response (considered a brisk jerk or jump or licking the affected area) was followed by the next smaller filament. The experimenter continued to move up or down the series, depending on the previously elicited response, for an additional four applications after the first positive response was observed for a minimum of five or a maximum of nine total monofilament applications. The value in log units of the final von Frey monofilament applied in the trial series was used to calculate a 50% g threshold for each mouse and group means were determined as previously described (Chaplan et al., 1994).

#### Micturition Analysis

For baseline, 1-day post-, and 8-day post-WAS micturition analysis, surfaces were cleaned with 70% ethanol and wiped dry. Naïve and NMS mice were acclimated in home cages to the testing room for 30 min prior to micturition pattern data collection. Mice were singly confined to a sheet of filter paper (11 cm × 5 cm × 3.5 cm) for 1 h using an inverted Micro-Isolator cage bottom (Lab Products Inc., Seaford, DE, United States). At the end of the testing period, the filter paper was left to dry while mice were returned to home cages and the animal facility. Once the filter papers were dry, fecal pellets were counted and urine spots were visualized using UV light. The total area (cm^2^) and number of urine spots were measured and recorded (NIH Image J). Separate groups of mice were tested at each time point to avoid potential confounds due to exposure to a novel environment.

#### Sucrose Preference Testing

Mice were individually housed in BioDAQ Liquid Choice Unplugged Allentown cages (Biological Data Acquisition; Research Diets, Inc., New Brunswick, NJ, United States) equipped with two Polysulfone BioDAQ drinking bottles. Following a 24-h acclimatization period during which both bottles contained standard drinking water, one bottle was filled with 1% sucrose diluted in drinking water and fluid volume levels were recorded and the position of the bottles interchanged daily for the duration of the experiment. The total volume of 1% sucrose consumed, as well as the percentage of 1% sucrose consumed was calculated on a daily basis.

### Acidified Toluidine Blue Mast Cell Staining

Mast cells were stained and quantified as previously described (Fuentes et al., 2015). Mice were overdosed with inhaled isoflurane (>5%) and intracardially perfused with ice cold 4% paraformaldehyde. Urinary bladders and prostates were removed, post-fixed in paraformaldehyde at room temperature for 1 h, cryoprotected in 30% sucrose in 1× PBS at 4°C overnight, and frozen in a heptane bath on dry ice. Tissue was transversely cut into 10 μm-thick (bladder) and 7 μm-thick (prostate) cryosections using a cryostat held at -22°C. Cryosections were then stained with acidified toluidine blue to visualize mast cells. Digital images of 8 separate, non-serial sections spanning the length of each tissue were captured (QIClick digital CCD Camera, Q Imaging, Surrey, BC, Canada) under light microscopic conditions (Nikon eclipse 90i, Nikon Instruments, Inc., Melville, NY, United States). The total number of non-degranulated mast cells (dense metachromasia with no evidence of granular extrusion) and degranulated mast cells (faint metachromasia, evident nucleus, extruded granules) were counted and the percentage of degranulated/total mast cells was calculated for each sample.

### mRNA Extraction and qRT-PCR

Mice were overdosed with inhaled isoflurane (>5%). Brains were removed and frozen on dry ice. Hypothalamus, amygdala, and hippocampus were dissected, immediately snap frozen in liquid nitrogen, and stored at -80°C. Brains were dissected, ventral-side up, on an inverted petri dish cleaned with 70% ethanol over a pack of wet ice. Brains were gently secured using forceps gripping the cerebellum/brain stem. The olfactory bulb and the frontal cortex were first removed and discarded by making a vertical cut in the frontal plane using a razor blade at the optic chiasm. Using a razor blade, the section containing the hypothalamus was removed from the rest of the brain by making a diagonal cut from the pons to the dorsal edge of the first cut, roughly a 45-degree angle. The temporal lobe on either side of the midbrain and the tissue dorsal to the hypothalamus, visually indicated by the anterior commissure for orientation, were trimmed away. From the remaining brain, a vertical cut was made at the rostral end of the pons and the cerebellum and brain stem were discarded. This interior section of brain was repositioned so that the surface of the frontal plane was against the glass of the petri dish. The amygdala was removed from the ventral corners of this section. Lastly, the hippocampus, visible on the side where the diagonal cut was made, was dissected by teasing away the cortex and the tissue inferior to the hippocampus using a scalpel. The urinary bladder was removed, bisected longitudinally [to facilitate both mRNA and protein (see below) analysis], snap frozen in liquid nitrogen, and stored at -80°C. The prostate was also removed, snap frozen, and stored similarly. Total RNA was isolated from dissected tissues using Trizol reagent (Ambion Inc., Austin, TX, United States) and RNeasy Mini Kit (Qiagen, Valencia, CA, United States). The concentration and purity was determined using a 2100 Bioanalyzer (Agilent Technologies, Santa Clara, CA, United States) and cDNA was synthesized from total RNA using the iScript cDNA Synthesis Kit (Bio-Rad, Hercules, CA, United States). Quantitative RT-PCR was performed using SsoAdvanced SYBR Green Supermix (Bio-Rad) and a Bio-Rad iCycler IQ real time PCR system with indicated 20 μM primers (Integrated DNA Technologies, Coralville, IA, United States). GAPDH was used as a housekeeping gene for brain tissues and its expression has previously been shown to be unaffected by acute or chronic stress exposure in PVN, hippocampus, and amygdala (Derks et al., 2008). β-actin was used as a housekeeping gene for bladder and prostate. Forward and reverse primer sequences for IL-6, IL-10, stem cell factor (SCF), artemin, NGF, MCP-1, CRF, Urocortin 2 (Ucn2), CRF_1_, CRF_2_, glucocorticoid receptor (GR), and mineralocorticoid receptor (MR) are reported in **Table 1**.

**Table 1:** RT-PCR forward and reverse primer sequences.

Gene	Forward (5′; – 3′;)	Reverse (3′; – 5′;)
IL-6	CTGCCAGAGACTTCCATCCAGTT	GAAGTAGGGAAGGCCGTGG
IL-10	GCTGGACAACATACTGCTAACC	ATTTCCGATAAGGCTTGGCAA
SCF	CCCTGAAGACTCGGGCCTA	CAATTACAAGCGAAATGAGAGCC
Artemin	GGCCAACCCTAGCTGTTCT	TGGGTCCAGGGAAGCTT
MCP-1	AGGTCCCTATGGTGCCAATGT	CGGCAGGATTTTGAGGTCCA
NGF	ACACTCTGATCACTGCGTTTTTG	CCTTCTGGGACATTGCTATCTGT
CRF	CCTCAGCCGGTTCTGATCC	GCGGAGGAAGTATTCTTCACCC
Ucn2	ACCCGTGTCATACTCTCCCTG	CAGCCTTGTAACGAGCCTG
CRF_1_	CCCTGCCTTTTTCTACGGTGT	TTCCCGGTAGCCATTGTTTGT
CRF_2_	CCTGTGGACACTTTTGGAGCA	TGTTGCAGTAGGTGTAGGGAC
GR	GACTCCAAAGAATCCTTAGCTCC	CTCCACCCCTCAGGGTTTTAT
MR	GAAAGGCGCTGGAGTCAAGT	CCATGTAGCTGTTCTCATTGGT
β-actin	AGTGTGACGTTGACATCCGTA	GCCAGAGCAGTAATCTCCTTCT
GAPDH	ATGTGTCCGTCGTGGATCTGA	ATGCCTGCTTCACCACCTTCTT

### Western Blot

Total protein was isolated from approximately 50 mg of snap-frozen bladder tissue using Cell Extraction Buffer (Invitrogen, Grand Island, NY, United States) containing Halt protease and phosphatase inhibitors (Thermo Fisher Scientific, Waltham, MA, United States) and Na_3_VO_4_ (Sigma, St. Louis, MO, United States). Protein concentrations were determined using a D_C_ protein assay (Thermo Fisher). Samples were reduced by heating to 95°C for 5 min in the presence of 2-mercaptoethanol, subjected to SDS–PAGE (Criterion 4–12% Bis-Tris gels; Bio-Rad, Hercules, CA, United States), and transferred to Nitrocellulose transfer membrane (Whatman GmbH, Dassel, Germany) by Criterion Blotter wet transfer (Bio-Rad). The membranes were blocked for 1 h at room temperature in 5% milk in tris-buffered saline with Tween-20 (TBST) and incubated overnight in 4°C with antisera to CRF_1_ (1:500; EMD Millipore, Billerica, MA, United States), CRF_2_ (1:800; Millipore), and GAPDH (1:2000; Cell Signaling Technology, Danvers, MA, United States) diluted in 5% milk in TBST. Membranes were then washed with TBST and incubated for 1 h with anti-rabbit secondary antibody (1:10,000; Cell Signaling). Densitometry was performed using Quantity One 4.6.9 software (Bio-Rad). We have previously used the CRF_1_ and CRF_2_ antisera in Western blot of bladder, vagina, and/or colon of female NMS mice (Pierce et al., 2014, 2015). These antisera have also been used in rat colon to determine changes due to NMS (O’Malley et al., 2010a,b). Per the manufacturer, both antibodies have been validated for immunohistochemistry and are specific for the N-terminal extracellular domain of the receptors with no reported cross-reactivity. We are reporting on the 65 kD band of CRF_1_, as this has been reported to most likely represent the fully glycosylated form of the receptor (Pisarchik and Slominski, 2004).

### Serum Corticosterone Enzyme-Linked Immunosorbent Assay (ELISA)

Trunk blood was collected at the time of sacrifice during the early half of the light-cycle (0800–1100 h), immediately following behavioral assessment, allowed to clot for 1 h on ice, and centrifuged at 10,000 rpm for 10 min. Serum (clear supernatant) was collected and stored at -20°C until analysis. Serum corticosterone (CORT) was quantified using enzyme-linked immunosorbent assay (ELISA) kit according to manufacturer’s instructions (ALPCO, Salem, NH, United States).

### Statistics

Calculations were made using Microsoft Excel. Normality of distribution was tested using Shapiro–Wilk’s test (*p* > 0.05) and measuring skewness and kurtosis. Non-normally distributed data was log transformed prior to statistical analysis. Statistical analysis was performed using Mann–Whitney *U* test, multiple regression, or two-way (with or without repeated measures) analysis of variance (ANOVA) followed by Fisher’s least significant difference (LSD) or Bonferroni’s posttest (IBM SPSS Statistics, IBM Corporation, Armonk, NY, United States; GraphPad Prism, GraphPad Software, La Jolla, CA, United States), as denoted in the manuscript. All data are expressed as mean ± SEM. A *p*-value of less than 0.05 was considered significant.

## Results

### Perigenital Hypersensitivity and Micturition

**FIGURE 1:**
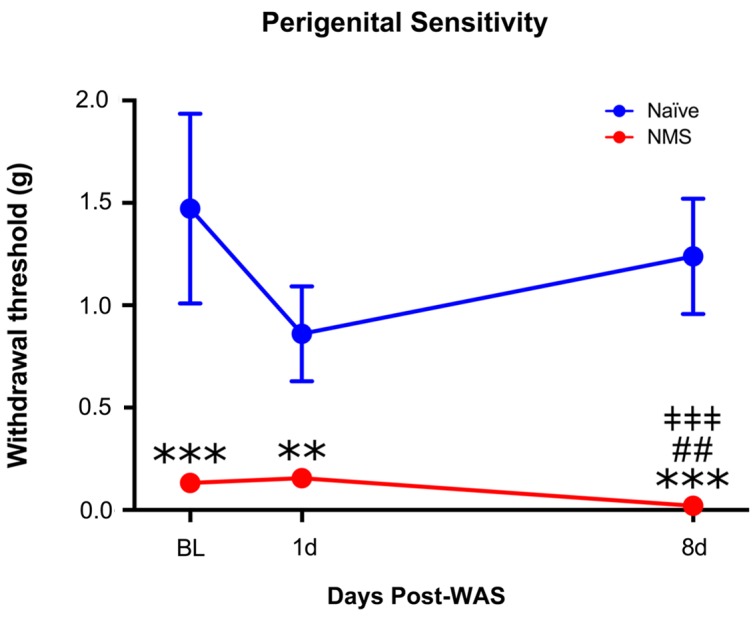
The impact of neonatal maternal separation (NMS) and water avoidance stress (WAS) on perigenital mechanical sensitivity. A significant impact of NMS (*p* < 0.0001, two-way RM ANOVA), WAS (*p* < 0.05), and an interaction effect of NMS and WAS (*p* < 0.01) was observed on mechanical withdrawal threshold. Exposure to NMS significantly reduced withdrawal thresholds at every time point, compared to naïve, which was further exacerbated at the 8-day post-WAS time point. ^∗∗^*p* < 0.01, ^∗∗∗^*p* < 0.001 vs. naïve, ^##^*p* < 0.01 vs. baseline, ^‡‡‡^*p* < 0.001 vs. 1-day post-WAS; Fishers’s least significant difference (LSD). *n* = 6 per group.

Adult naïve and NMS mice were assessed for perigenital mechanical sensitivity prior to and following exposure to WAS. Exposure to NMS significantly reduced mechanical withdrawal thresholds [*F*(1,10) = 102.1, *p* < 0.0001, two-way RM ANOVA], as did exposure to WAS [*F*(2,20) = 4.159, *p* = 0.0309] (**Figure 1**). An additional interaction effect of NMS and WAS [*F*(2,20) = 6.608, *p* = 0.0063] was also observed. *Post hoc* analysis revealed a significant decrease in withdrawal threshold of NMS mice at all time points, compared to naïve (*p* < 0.01, Fisher’s LSD), and at 8-day post-WAS compared to prior measurements (*p* < 0.01) (**Figure 1**).

**FIGURE 2:**
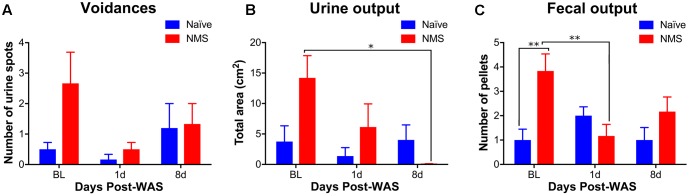
The impact of NMS and WAS on micturition patterning and fecal output. **(A)** The number of voids was significantly impacted by NMS and WAS, at the 1-day post-WAS time point, as determined by multiple regression analysis (*p* < 0.05, *R*^2^ = 0.318). **(B)** Total urine output was also significantly impacted by NMS and WAS at the 1-day post-WAS time point *(p* < 0.05, *R*^2^ = 0.310), with an additional significant decrease at 8-day post-WAS, compared to baseline, in NMS mice. **(C)** Fecal output was significantly impacted by NMS (*p* < 0.05, two-way ANOVA) and a NMS and WAS interaction effect (*p* < 0.01). Baseline fecal output was significantly higher in NMS mice compared to both naïve baseline output and that of 1-day post-WAS NMS mice. Brackets indicate significant effects: ^∗^*p* < 0.05, ^∗∗^*p* < 0.01; **(B)** Mann-Whitney *U* test; **(C)** Bonferroni posttest. *n* = 6 per group.

In addition to mechanical sensitivity, mice were assessed for micturition frequency and output. Multiple regression analysis was calculated to predict urine output based on NMS and WAS exposure. A significant regression equation was found for micturition frequency [*F*(2,21) = 9.375, *p* = 0.018], with an *R*^2^ of 0.318 at the 1-day post-WAS time point, but not at the 8-day post-WAS time point (**Figure 2A**). Both NMS and WAS were significant predictors of micturition frequency (*p* = 0.038). A significant regression equation was also found for total urine output [*F*(2,21) = 4.723, *p* = 0.020], with an *R*^2^ of 0.310 at 1-day post-WAS, but not 8-day post-WAS (**Figure 2B**). Only NMS exposure was a significant predictor of total urine output (*p* = 0.019). Exposure to NMS [*F*(2,30) = 5.957, *p* = 0.0208] significantly increased fecal output and a significant interaction effect of NMS and WAS [*F*(2,30) = 6.007, *p* = 0.0064] was also observed (**Figure 2C**). *Post hoc* analysis revealed a significant decrease in fecal output in NMS mice at 1-day post-WAS compared to baseline measurements (*p* < 0.01).

### Anhedonic Behavior

**FIGURE 3:**
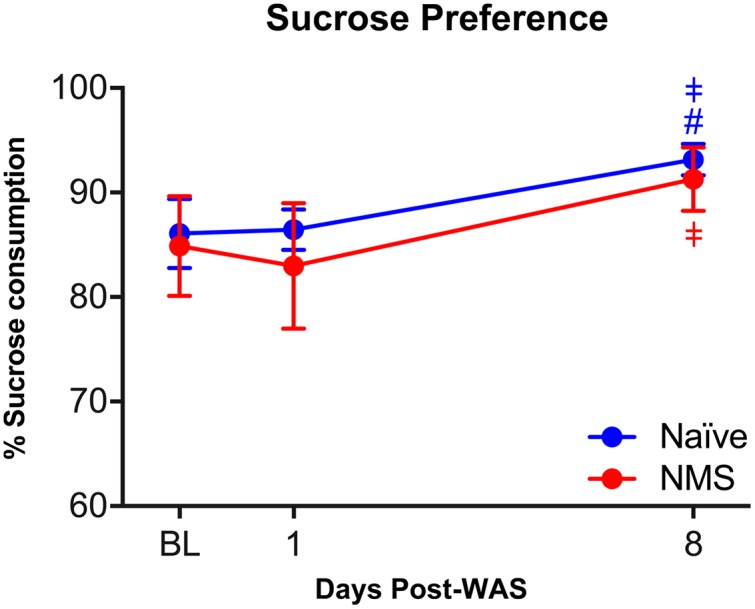
The impact of NMS and WAS on anhedonic behavior. Exposure to NMS had no impact on sucrose preference, however, exposure to WAS increased sucrose preference in both groups over time (*p* < 0.001, two-way RM ANOVA). *Post hoc* analyses revealed a significant increase in sucrose preference at 8-day post-WAS in both groups, compared to baseline and/or 1-day post-WAS consumption rates. ^#^*p* < 0.05 vs. baseline, ^‡^*p* < 0.05 vs. 1-day-post WAS; Fishers’s LSD. *n* = 5–6 per group.

Anhedonic behavior is a hallmark symptom of depression, which is a common comorbidity among CP/CPPS and IC/PBS patients (Rodriguez et al., 2009). Preference for 1% sucrose was measured in naïve and NMS mice prior to and following exposure to WAS. No differences in sucrose preference were observed between naïve and NMS mice at baseline (**Figure 3**). No differences were observed between groups following WAS exposure; however, both groups had a significant increase in sucrose preference over time [*F*(2,18) = 11.22, *p* = 0.0007] and at 8-day post-WAS (*p* < 0.05; **Figure 3**), compared to relative baseline and 1-day post-WAS preferences.

### Mast Cell Degranulation in Urogenital Tissues

**FIGURE 4:**
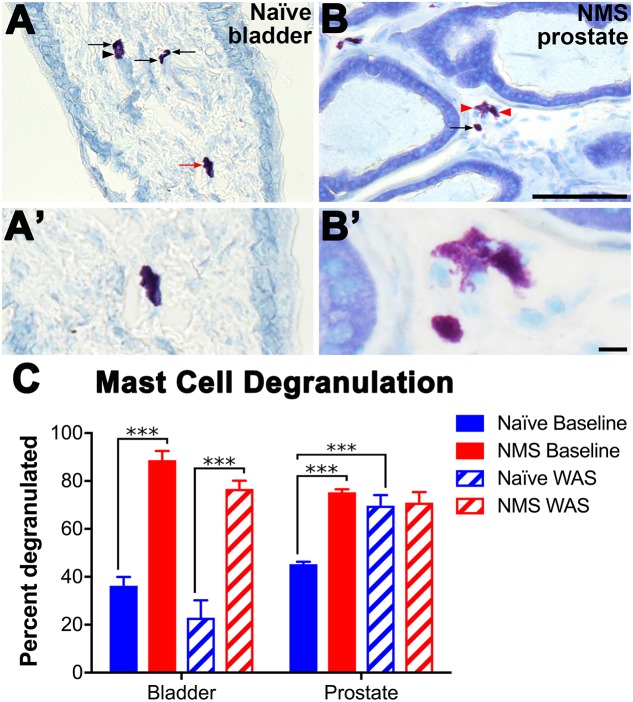
Histological identification of mast cell degranulation in urogenital tissues. **(A–B′;)** Photomicrographs show intact (arrow) and degranulated (arrowhead) mast cells visualized in naïve bladder **(A,A′;)** and NMS prostate **(B,B′;)** tissue samples. Higher magnification reveals that non-degranulated mast cells could be identified by intact cell membranes and dark purple color (**A**′;, red arrow **A**), while degranulated mast cells typically stained a light purple, did not have intact cell membranes, and the granule contents were visible and/or appeared to burst from the cell (**B**′;, red arrowheads **B**). **(C)** Mast cell degranulation rates were significantly increased by NMS in both the bladder (*p* < 0.0001, two-way ANOVA) and prostate (*p* < 0.001) and differentially decreased and increased by WAS in the bladder and prostate, respectively (*p* < 0.05). Significant differences between naïve and NMS were observed at baseline in both tissues and WAS had no impact on naïve bladder degranulation rates, whereas it significantly increased degranulation in naïve prostate, compared to naïve baseline rates. Brackets indicate significant differences: ^∗∗∗^*p* < 0.001; Bonferroni’s posttest. *n* = 3–4 per group. Scale bars represent 100 μm **(A,B)** and 10 μm **(A′;,B′;)**. *n* = 3–4 per group.

Acidified toluidine blue was used to histologically identify and determine degranulation states of resident mast cells in bladder and prostate tissues from naïve and NMS mice not exposed to WAS (baseline) and 8 days after WAS exposure. Mast cells were readily apparent, and intact versus partially degranulated mast cells were easily distinguishable (**Figures 4A–B′;**). Both WAS [*F*(1,11) = 6.411, *p* = 0.0279] and NMS [*F*(1,11) = 112.2, *p* < 0.0001] had significant main effects on bladder mast cell degranulation. At baseline, both bladder and prostate from NMS mice displayed a significant increase in the percentage of mast cells with evidence of degranulation, compared to tissues from naïve mice (*p* < 0.0001; **Figure 4C**). Eight days after WAS exposure, mast cell degranulation rates remained unchanged in naïve and NMS bladder (**Figure 4C**). WAS [*F*(1,11) = 8.275, *p* = 0.0151] and NMS [*F*(1,11) = 20.06, *p* = 0.0009] had simple main effects and an interaction effect [*F*(1,11) = 17.14, *p* = 0.0016] on prostatic mast cell degranulation. At baseline, NMS prostate had a significantly higher mast cell degranulation rate than naïve prostate (*p* < 0.001); however, WAS exposure in naïve mice significantly increased degranulation rates compared to baseline measurements (*p* < 0.001; **Figure 4C**).

**FIGURE 5:**
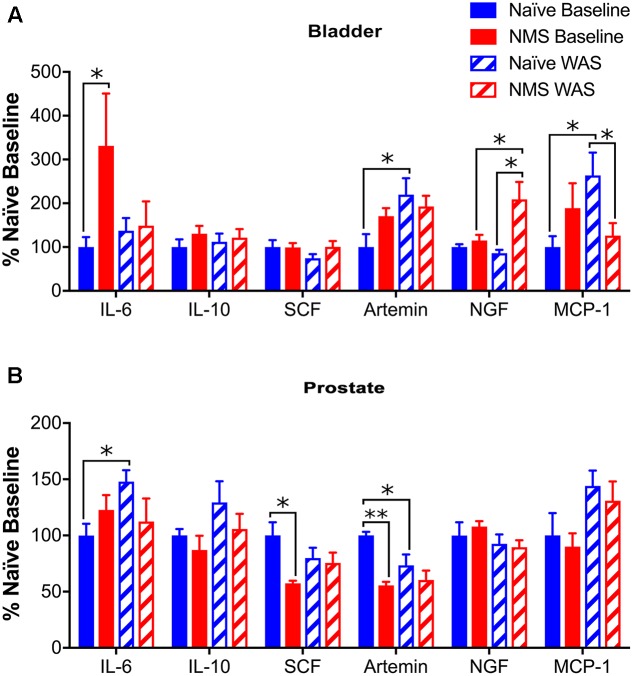
Cytokine and growth factor mRNA levels in urogenital tissues. **(A)** A significant increase in IL-6 mRNA levels was observed in NMS bladder, compared to naïve, at baseline. Artemin mRNA levels were significantly increased by WAS (*p* < 0.05, two-way ANOVA), particularly in naïve-WAS bladders compared to baseline. Levels of NGF mRNA were significantly impacted by NMS (*p* < 0.01) and a NMS/WAS interaction (*p* < 0.05), resulting in significantly higher levels in NMS–WAS bladder, compared to both NMS-baseline and naïve-WAS. Levels of MCP-1 mRNA were also impacted by a NMS/WAS interaction (*p* < 0.05), with significantly higher expression in naïve-WAS compared to naïve-baseline and NMS–WAS levels. No change in mRNA levels was observed for either IL-10 or SCF in the bladder. **(B)** In the prostate, IL-6 was significantly increased in naïve-WAS compared to baseline levels. The levels of SCF and artemin were both significantly decreased by NMS (*p* < 0.05), with an additional NMS/WAS interaction effect on SCF levels (*p* < 0.05). The levels of MCP-1 mRNA were significantly increased by WAS (*p* < 0.05). No significant effect of NMS and/or WAS was observed for IL-10 or NGF in the prostate. All values are expressed as a percentage of naïve baseline measurements for each gene/tissue. Brackets indicate significant effects: ^∗^*p* < 0.05, ^∗∗^*p* < 0.01; Fisher’s LSD. *n* = 5–6 per group.

To determine the effect of NMS and WAS on expression of genes involved in mast cell recruitment and activation, RT-PCR was used to measure IL-6, IL-10, SCF, artemin, NGF, and MCP-1 mRNA levels in the bladder and prostate. In bladder, IL-6 mRNA levels were significantly higher in NMS than naïve at baseline (*p* < 0.05). WAS had a significant effect on increasing artemin mRNA levels [*F*(1,18) = 5.850, *p* = 0.0264; **Figure 5A**]. Increases in NGF mRNA levels were significantly affected by NMS [*F*(1,18) = 8.862, *p* = 0.0081], as well as an NMS/WAS interaction effect [*F*(1,18) = 5.427, *p* = 0.0317; **Figure 5A**]. NMS–WAS males displayed the greatest increase of NGF mRNA levels; significantly greater than both naïve and NMS controls (*p* < 0.05). An NMS/WAS interaction effect also had a significant impact on increased MCP-1 mRNA levels [*F*(1,18) = 6.830, *p* = 0.0176; **Figure 5A**]. Similar to bladder values, prostatic IL-6 and IL-10 were not statistically altered by NMS or WAS exposure (**Figure 5B**). Expression of SCF was not altered in bladders across all four groups (**Figure 5A**); however, in the prostate, decreased SCF mRNA levels were attributed to NMS [*F*(1,16) = 7.256, *p* = 0.0160] and NMS/WAS interaction effects [*F*(1,16) = 4.854, *p* = 0.0426; **Figure 5B**]. Artemin mRNA levels were significantly decreased by NMS [*F*(1,16) = 14.31, *p* = 0.0016] and unaffected by WAS exposure (**Figure 5B**). Exposure to WAS significantly increased MCP-1 mRNA levels in the prostate [*F*(1,16) = 7.110, *p* = 0.0184; **Figure 5B**].

### Limbic Regulation of the HPA Axis

**FIGURE 6:**
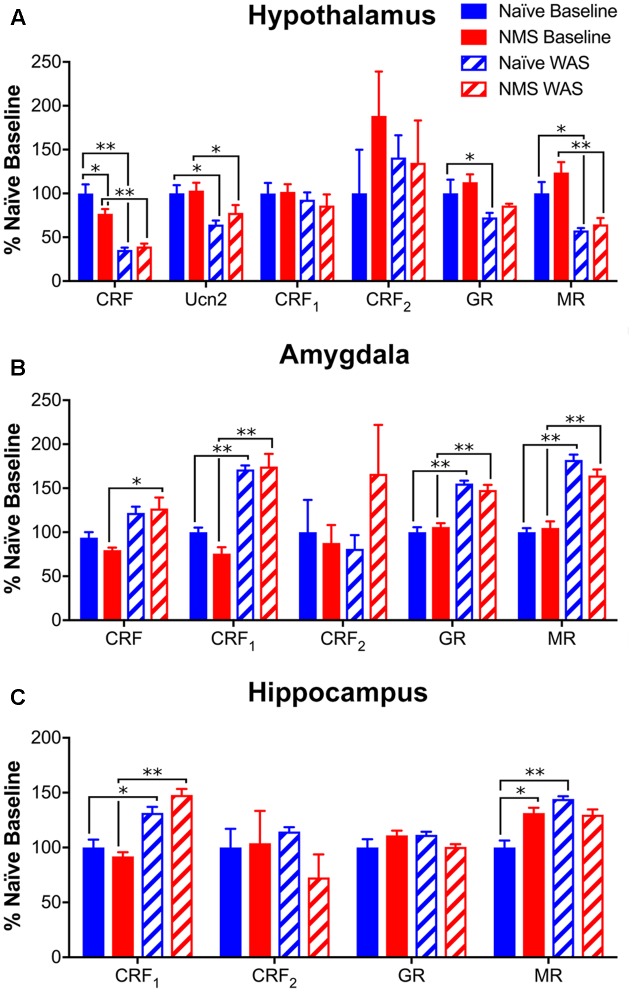
Glucocorticoid- and corticotropin releasing-factor (CRF)-related gene expression levels in central brain regions. **(A)** In the hypothalamus, mRNA levels of both CRF and Ucn2 were significantly decreased by NMS (*p* < 0.01, two-way ANOVA) with an additional NMS/WAS interaction effect on CRF (*p* < 0.05). Baseline NMS CRF mRNA levels were also significantly lower than naïve. The mRNA levels of glucocorticoid receptor (GR) and mineralocorticoid receptor (MR) were also significantly reduced by WAS exposure (*p* < 0.01). No significant effect of NMS and/or WAS was observed on CRF1 or CRF2 mRNA levels in the hypothalamus. **(B)** In the amygdala, WAS significantly increased the mRNA levels of CRF, CRF1, GR, and MR (*p* < 0.01), with no significant effect observed on CRF2 mRNA levels. **(C)** In the hippocampus, WAS significantly increased CRF1 mRNA levels (*p* < 0.0001). A NMS/WAS interaction effect was observed on GR mRNA levels (*p* < 0.05) and MR mRNA levels were significantly impacted by WAS and a NMS/WAS interaction (*p* < 0.05). Baseline MR mRNA levels were significantly higher in NMS compared to naïve. All values are expressed as a percentage of naïve baseline measurements for each gene/tissue. Brackets indicate significant effects: ^∗^*p* < 0.05, ^∗∗^*p* < 0.01; Fisher’s LSD. *n* = 5–6 per group.

To determine the impact of NMS and WAS on gene expression within central structures involved in the regulation and output of the HPA axis, mRNA levels of CRF, Ucn2, CRF_1_, CRF_2_, GR, and MR in hypothalamus, amygdala, and hippocampus of mice were determined by RT-PCR. In the hypothalamus, NMS and WAS had a significant interaction effect on CRF mRNA levels [*F*(1,17) = 5.184, *p* = 0.0360; **Figure 6A**]. Exposure to WAS significantly decreased hypothalamic CRF [*F*(1,17) = 74.06, *p* < 0.0001], Ucn2 [*F*(1,18) = 14.16, *p* = 0.0014], GR [*F*(1,17) = 8.74, *p* = 0.0088], and MR [*F*(1,18) = 31.34, *p* < 0.0001] mRNA levels in naïve and NMS mice, with no effect of NMS or WAS observed on either CRF receptor (**Figure 6A**). In contrast, exposure to WAS significantly increased CRF [*F*(1,18) = 10.33, *p* = 0.0048], CRF_1_ [*F*(1,18) = 85.11, *p* < 0.0001], GR [*F*(1,18) = 97.76, *p* < 0.0001], and MR [*F*(1,18) = 120.9, *p* < 0.0001] mRNA levels in the amygdala (**Figure 6B**). In the hippocampus, exposure to WAS significantly increased CRF_1_ [*F*(1,17) = 25.82, *p* < 0.0001] and MR [*F*(1,17) = 5.374, *p* = 0.0332] expression across both naïve and NMS mice (**Figure 6C**). A significant interaction effect of NMS and WAS was also observed for MR [*F*(1,17) = 13.42, *p* = 0.0019], as well as for GR [*F*(1,17) = 5.789, *p* = 0.0278] mRNA levels in the hippocampus (**Figure 6C**).

### Peripheral CRF Receptor Expression

**FIGURE 7:**
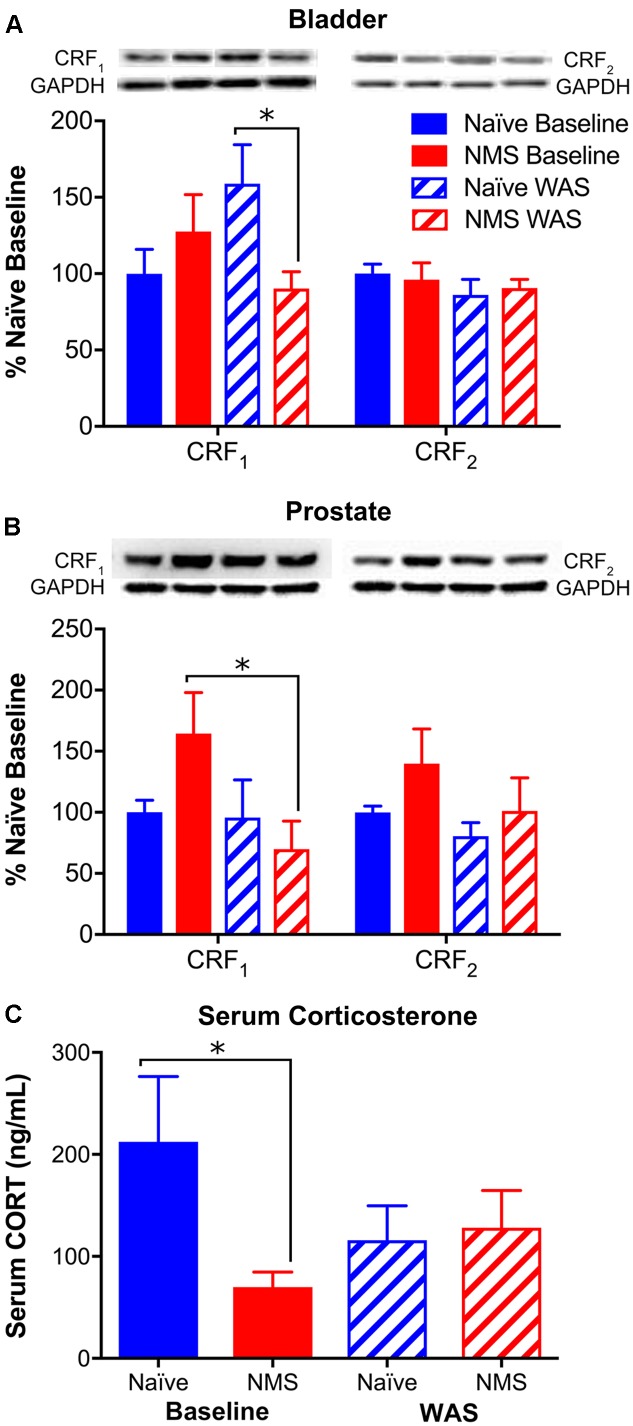
Protein levels of CRF receptors in urogenital organs and CORT in serum. **(A,B)** Representative Western blots are shown for CRF_1_, CRF_2_, and corresponding GAPDH protein with bands at 65, 49, and 35 kD, respectively. Bands representing each group correspond with the mean values represented underneath and may not have been contiguous within the original gel. The GAPDH bands in **(B)** are repeated, as these correspond to both CRF_1_ and CRF_2_ in the same samples, as shown in detail in Supplementary Figure 1. **(A)** A significant interaction effect of NMS and WAS was observed on CRF_1_ protein levels in the bladder (*p* < 0.05, two-way ANOVA), resulting in significantly lower expression in NMS–WAS compared to naïve-WAS. No effect of NMS and/or WAS was observed for CRF2 protein levels in the bladder. **(B)** In the prostate, CRF_1_ protein levels were significantly lower in*(NMS–WAS compared to NMS-baseline. No effect of NMS and/or WAS was observed on CRF_2_ protein levels in the prostate. **(C)** Serum CORT protein levels were significantly lower in NMS mice at baseline, compared to naïve. No significant effect of WAS was observed on serum CORT protein levels. Brackets indicate significant effects: ^∗^*p* < 0.05; Fisher’s LSD. *n* = 5–6 per group.)*

To complement our central HPA axis-associated mRNA level investigation, we used Western blot to measure peripheral CRF_1_ and CRF_2_ protein expression, as well as ELISA analysis to measure serum CORT concentrations following NMS and/or WAS. Bladder CRF_1_ protein expression was significantly influenced by an NMS/WAS interaction [*F*(1,17) = 6.154, *p* = 0.0239], resulting in significantly lower CRF_1_ protein in NMS–WAS bladder than naïve-WAS (**Figure 7A**). Prostatic CRF_1_ protein was significantly lower in NMS–WAS compared to baseline measurements (**Figure 7B**). CRF_2_ protein levels, on the other hand, were not significantly altered in either the bladder or the prostate across all four groups (**Figures 7A,B**). Serum CORT levels were significantly lower in baseline NMS males compared to naïve counterparts (**Figure 7C**). WAS-exposed groups displayed similar concentrations, but were not significantly different from baseline levels (**Figure 7C**).

## Discussion

Early life stress or experiences of adverse childhood events have been associated with improper functioning of the HPA axis and can serve as risk factors for functional pain disorders in adulthood (Hu et al., 2007; Tyrka et al., 2008; Videlock et al., 2009; Riegel et al., 2014). Here, we have provided evidence that early life stress is capable of inducing perigenital mechanical hypersensitivity and increased urinary bladder output, combined with histological evidence of enhanced mast cell degranulation in the bladder and prostate of male mice. Exposure to adult stress increased perigenital sensitivity, but not urine output, of NMS mice and altered neuroimmune profiles in both bladder and prostate and gene expression within the hypothalamus and limbic regions. These data suggest that early life stress contributes to a painful phenotype that is variably affected by acute stress in adulthood.

Clinically, CP/CPPS and IC/PBS are characterized by chronic, idiopathic pelviperineal referable pain with or without urinary symptoms such as urgency, frequency, and nocturia (Litwin et al., 1999; Baranowski et al., 2008). As in a previous study (Fuentes et al., 2015), male NMS mice displayed significantly lower perigenital mechanical withdrawal thresholds compared to naïve controls at baseline. Acute exposure to WAS did not significantly impact withdrawal thresholds of naïve mice; however, the NMS mice had significantly lower thresholds at 8-day post-WAS compared to their previous measurements. Micturition frequency, total urine output, and fecal output were all increased in NMS mice at baseline. Interestingly, exposure to WAS decreased all of these measures, suggesting that exposure to novel acute stress normalized both bladder and colon output. Different groups of mice were tested at each time point to control for the potential anxiogenic effect of exposure to a novel environment, which could affect marking behaviors. The lack of negative impact of WAS on baseline perigenital sensitivity and micturition in male NMS mice is the exact opposite of what we observed in female NMS mice, which demonstrated significantly increased bladder sensitivity at 8-day post-WAS and transiently increased micturition frequency and output at 1-day post-WAS (Pierce et al., 2016).

Patients with CP/CPPS and IC/PBS commonly suffer from mood disorders (Rodriguez et al., 2009), and we previously observed an increase in anxiety-like behavior following acute stress exposure in male (Fuentes et al., 2016) and female (Pierce et al., 2014) NMS mice. In this study, we investigated potential anhedonic behavior by measuring sucrose preference. While no differences in sucrose preference were observed between NMS and naïve mice, exposure to WAS increased sucrose preference in both groups of mice over time indicating reduced anhedonic behavior. These observations suggest that despite prior evidence of increased anxiety-like behavior, NMS and WAS do not impact depression-like behaviors, and, if anything, WAS exposure increased the drive for pleasure-seeking behavior over time.

Mast cells have been a major focus of research in CP/CPPS, as well as other functional pain disorders (Murphy et al., 2014). Previous work from our lab has shown an increase in mast cell degranulation in urogenital organs of both male (Fuentes et al., 2015) and female (Pierce et al., 2016) NMS mice, as well as an increase in mast cell degranulation in naïve bladder following WAS exposure in the latter study. Here, we again observed an increase in mast cell degranulation in both bladder and prostate of NMS mice, as well as an increase in degranulation following WAS in naïve prostate. Changes in mRNA levels related to mast cell infiltration and activation were also observed. The mRNA levels of MCP-1, a known mast cell activator (Conti and DiGioacchino, 2001), were significantly increased in both naïve prostate and bladder following WAS, and increased IL-6 mRNA levels in naïve prostate post-WAS may be related to the increased mast cell degranulation in the same tissue. Interestingly, SCF, which attracts mast cell precursors and initiates their differentiation by binding to the receptor c-kit (Draber et al., 2016), was unchanged in the bladder and significantly decreased by NMS and WAS in the prostate, suggesting that although degranulation rates had increased, recruitment of mast cells to the affected tissue is not being increased via a SCF-mediated mechanism. Artemin and NGF mRNA levels were both increased in the bladder following WAS exposure, particularly in NMS bladder; however, these levels were either decreased or unaffected by NMS and WAS in the prostate. NGF can be released from mast cells and contribute to a feed-forward degranulation process (Done et al., 2012), as well as sensitize sensory nerves and contribute toward increased pain in CP/CPPS (Horigome et al., 1993; Done et al., 2012). Mast cell tryptase and NGF have been considered the most promising biomarkers of CP/CPPS, and the prominent role of NGF in IC/PBS further strengthens the hypothesis that CP/CPPS and IC/PBS are different manifestations of the same underlying disorder. Because of its role in generation and potentiation of pain following tissue injury and inflammation (Hefti et al., 2006), NGF has become a therapeutic target, yielding marginal success; therapeutic inhibition of NGF decreased pain-like behavior responses in a number of animal models of visceral pain (Jaggar et al., 1999), as well as IC/PBS in humans (Evans et al., 2011).

Exposure to NMS has generally been shown to increase the output of the HPA axis, evidenced as enhanced anxiety-like behaviors and prolonged release of ACTH and CORT following a stressful event (Romeo et al., 2003; Ladd et al., 2004; Plotsky et al., 2005; Aisa et al., 2008). Production of CRF in both the hypothalamus and the amygdala was increased in NMS rats at baseline, as well as following stress exposure (Plotsky et al., 2005; Aisa et al., 2008; Desbonnet et al., 2008). NMS has also been shown to affect limbic feedback through altered expression of CRF and GRs (Ladd et al., 2004; Aisa et al., 2008; O’Malley et al., 2011b). A recent study in mice, incorporating a genetic deletion of pituitary adenylate cyclase-activating polypeptide (PACAP), maternal separation, and adult stress exposure as a three-hit model of depression, demonstrated increased CRF-positive neurons in the bed nucleus of the stria terminalis (Farkas et al., 2017), an additional limbic structure that plays a significant role in stress response. In the current study, rather than increasing molecular evidence of HPA activation, WAS exposure significantly decreased CRF, Ucn2, GR, and MR mRNA expression in the hypothalamus across both naïve and NMS mice. The opposite trend was observed in the limbic structures, as WAS significantly increased CRF, CRF_1_, GR, and MR mRNA levels in the amygdala and CRF_1_ in the hippocampus across both groups. Both GR and MR were significantly affected by a NMS/WAS interaction, indicating divergent responses in gene expression to WAS between naïve and NMS mice. When these data are taken into consideration with the changes in peripheral CRF_1_ and lower serum CORT concentrations, we conclude that the NMS male mice may present with hypocortisolism, which has also been observed in children who were exposed to severe deprivation, neglect, or abuse (Gunnar and Quevedo, 2008; Lupien et al., 2009). In human studies, it has been hypothesized that lower basal glucocorticoid levels may be due to a downregulation of the HPA axis at the level of the pituitary in response to chronic drive of CRF from the hypothalamus (Fries et al., 2005), or target tissue hypersensitivity to glucocorticoids (Yehuda et al., 2006). Stress and/or chronic glucocorticoid exposure promotes CRF release in the central amygdala (Makino et al., 1994, 1999; Cook, 2004), suggesting that stress desensitization may be involved in the development of stress-related pathologies (Herman et al., 2012).

## Conclusion

We have tested the hypothesis that early life stress in male mice affects perigenital sensitivity, micturition patterning, and depression-like behavior, as well as possible exacerbation by acute adult stress. Exposure to NMS increased perigenital mechanical sensitivity and bladder output, which corresponded with increased mast cell degranulation and IL-6 mRNA expression in the urogenital organs. Central gene expression patterns, as well as significantly lower serum CORT levels, suggest that hypocortisolism may be driving these baseline changes. Following WAS exposure, the perigenital sensitivity was exacerbated in the NMS mice; however, urine output was restored to normal levels, suggesting a dichotomous effect of adult stress exposure on urogenital sensitivity and function. Receptor levels for CRF and glucocorticoids were increased within the amygdala and hippocampus following WAS exposure in both naïve and NMS mice, suggesting an increase in negative limbic regulation onto the hypothalamus, which may have influenced cytokine and growth factor mRNA levels in the urogenital tissues. This study illustrates the central, peripheral, and behavioral outcomes of early life and adult stress exposure and the potential future use of NMS in male mice as a preclinical model of male chronic urological pain syndromes for investigating pharmacological and interventional therapies.

## Author Contributions

JC designed the study. IF, AP, EDS, and MM collected the data. JC, IF, and AP analyzed and interpreted the data. JC and IF wrote the manuscript.

## Conflict of Interest Statement

The authors declare that the research was conducted in the absence of any commercial or financial relationships that could be construed as a potential conflict of interest.
