# Supplementary figures and images for: Differential Influence of Early Life and Adult Stress on Urogenital Sensitivity and Function in Male Mice
Source: Front Syst Neurosci. 2018 Jan 9;11:97. doi: 10.3389/fnsys.2017.00097 (PMC5771376; doi:10.3389/fnsys.2017.00097)

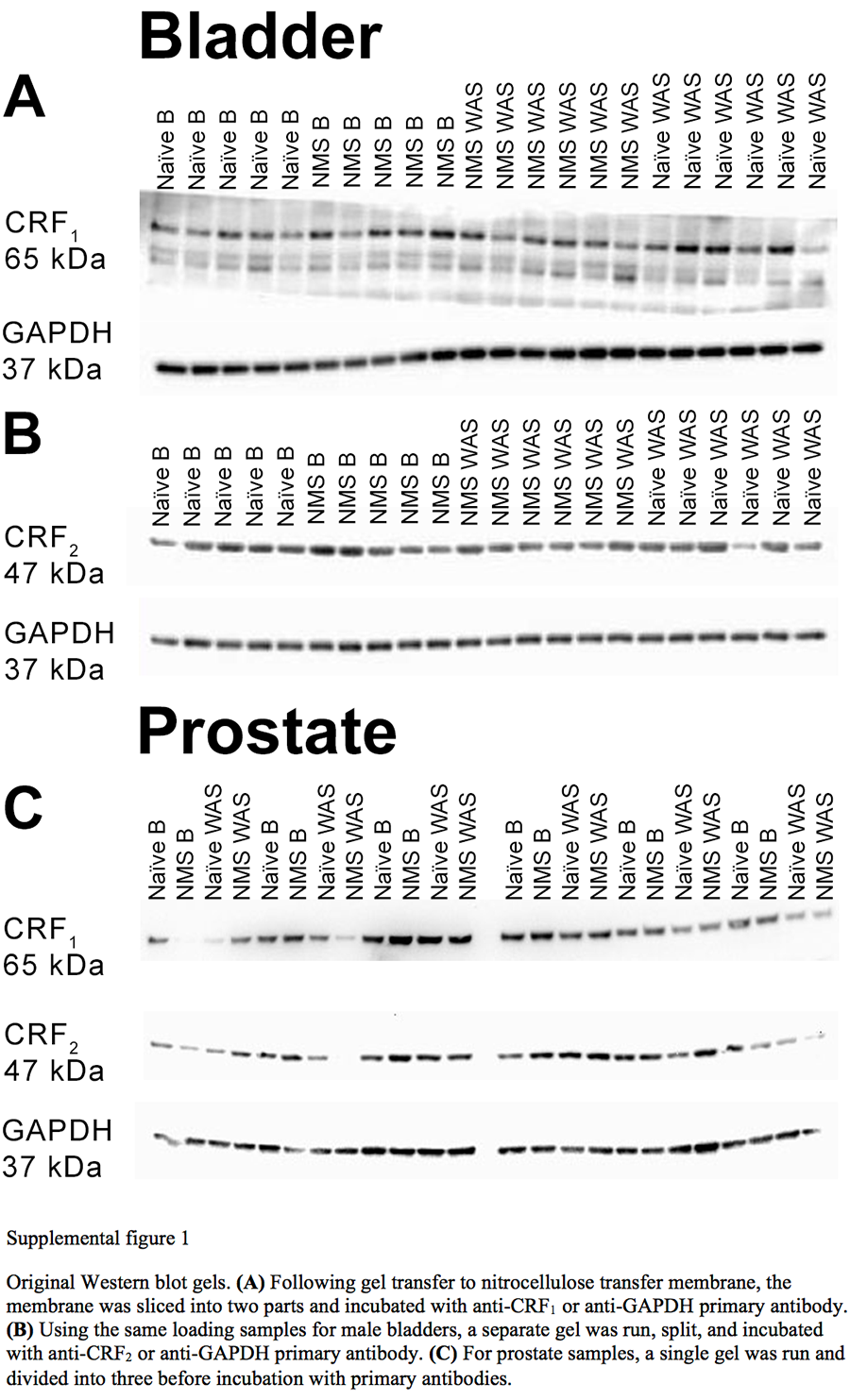

Supplement: Supplementary file 1 [file Image_1.TIF]
